# Variation of Crystal Orientation and Dendrite Array Generated in the Root of SX Turbine Blades

**DOI:** 10.3390/ma12244126

**Published:** 2019-12-09

**Authors:** Włodzimierz Bogdanowicz, Jacek Krawczyk, Robert Paszkowski, Jan Sieniawski

**Affiliations:** 1 Institute of Materials Engineering, University of Silesia in Katowice, 1A 75 Pulku Piechoty St., 41-500 Chorzów, Poland; wlodzimierz.bogdanowicz@us.edu.pl; 2 Department of Materials Science, Rzeszów University of Technology, 2 Wincentego Pola St., 35-959 Rzeszów, Poland; jansien@prz.edu.pl

**Keywords:** superalloy, X-ray topography, defects, lateral growth, dendrite array, low-angle boundaries

## Abstract

The variation of the crystal orientation and the dendrite array generated in the root of the single-crystalline (SX) turbine blades made of CMSX-4 superalloy were studied. The blades with an axial orientation of the [001] type were solidified by the industrial Bridgman technique using a spiral selector at a withdrawal rate of 3 mm/min. The analysis of the crystal orientation and dendrite arrangement was carried out using scanning electron microscopy, X-ray diffraction topography, and Laue diffraction. It was found that the lateral growth of such secondary dendrite arms, which are defined as “leading” and grow in the root at first, is related to the rotation of their crystal lattice, which is the reason for creation of the low-angle boundary (LAB) type defects. The primary crystal orientation of the selector extension (SE) area determines the areas and directions of the lateral growth of the leading arms. Additionally, it was found that in the SE areas of the root, near the connection with the selector, the spatial distribution of the [001]γ′ crystallographic direction has a complex wave-like character and may be related to the shape of the crystallization front.

## 1. Introduction

The nickel-based superalloys are the most widely used materials for production of single-crystalline (SX) turbines components, which is applicable in aerospace and energy sectors. The combination of good mechanical properties (i.e., high mechanical strength and creep resistance) and corrosion resistance at high temperature (e.g., the operating temperature is about 1150 °C for TMS238) make superalloys useful in the production of blades for high pressure turbines [1,2,3,4]. For example, the creep rupture lives of the representative superalloys is about 1000 h for the third-generation alloy, RR3000. The ultimate tensile strength UTS for TMS238 is 1348 MPa at 750 °C [1,3,5,6]. The required properties are met for the SX blades made of nickel-based CMSX-4 superalloy, in which the [001] crystallographic direction is parallel to the blade axis. The SX turbine blades are usually obtained in casting molds by dendritic directional crystallization using the Bridgman technique. The process leads to the obtainment of the SX blade casts, the structure and defects of which largely depend on the array and crystal orientation of the formed dendrites, and in turn affect the blades properties.

The CMSX-4 superalloy casts are mainly composed of the fcc γ-phase solid solution as a matrix, which is strengthened by precipitation of the intermetallic γ′-phase (Ni_3_Al) in the form of cubic crystals. The γ/γ′ microstructure is formed after the crystallization of γ dendrites by the phase transformation of solid solution. Therefore, the γ/γ′ microstructure is related to the crystal orientation and array of the dendrites. The complex shape of the blade cast affects the formation of the dendrite array and its crystal orientation, which is related to the γ/γ′ structure and cast defects. Some of these defects are not eliminated in subsequent stages of blades production, e.g., during heat treatment [7]. The crystal orientation, which depends on the parameters of the directional crystallization and the blades’ geometry [8,9,10], has a significant effect on the mechanical properties [1,11,12], such as stress rupture life and elongation of the SX superalloy casts [13]. Additionally, the presence and localization of the low-angle boundaries (LABs) in the blades, which are related to the cast geometry, affect the tensile strength and creep resistance [14].

**Figure 1:**
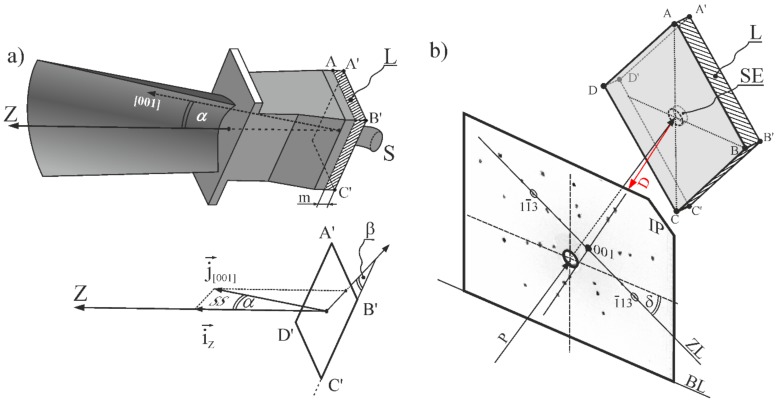
Scheme of the L sample location in the root and arrangement of (${\vec{i}}_{z}$ and ${\vec{j}}_{\left[001\right]}$ unit vectors (**a**) and the arrangement of Image Plate (IP) and sample during Laue pattern formation in reflection geometry (**b**). S—spiral selector; P—primary beam, D—diffraction beams; SE—selector extension area; ABCD and A’B’C’D’—surfaces of sample L; Z—axis of blade; ${\vec{i}}_{z}$ and ${\vec{j}}_{\left[001\right]}$—unit vectors parallel to the Z and [001]; SS—plane of [001] inclination; BL—base line of the lauegram. The other symbols are descripted in the text.

To set up the [001] primary orientation of the SX blade axis during crystallization by the Bridgman technique, the spiral selector (common called as “pigtail”) is mostly used; however, the secondary orientation remains random. The value of the inclination angle of the [001] direction (α-Figure 1a) relative to the Z-axis as well as the arrangement of the SS plane of this inclination relative to one of the edges (B’C’) of the blade root are also random. The arrangement of the plane is defined by the β angle (Figure 1a). Both the α and β angles have a stochastic character. The local changes in the crystal orientation are related to the local changes in the direction of dendrite growth, while for alloys with an fcc structure, it is commonly assumed that the dendrites grow strictly along the [001]-type crystallographic directions [15]. The interaction of the dendrites with tilted mold walls may cause a deviation from the preferred crystal orientation during crystallization [16]. Even small local differences (dozens of arc minutes) in the local crystal orientation cause the creation of the LABs during casting, or later, during heat treatment, reducing the strength [14]. This effect depends on the values of the LAB misorientation angle [14,17].

In the SX blades, one of the planar type defects of misorientation character is that of the LABs. They may be created during the transition of the crystallization front from the selector to the root and inherited by the entire turbine blade [18]. The creep resistance of the blades is related to the LABs misorientation angle [14]. When the LAB misorientation increased, the elongation decreased at temperatures above 850 °C. It is notable that the effects of the LAB on the ultimate tensile strength are weaker than those on the tensile elongation [14]. Therefore, it is extremely important to study the root area located near the selector. The above-mentioned defects cause a local increase in the number of dislocations and point defects [19], which can affect the formation of the γ/γ′ structure after subsequent heat treatment of the SX blades. The concentration of the vacancy-type defects and dislocations in multicomponent CMSX-4 superalloy containing many alloying elements is particularly important and related to the kinetics of the diffusion processes [20,21], on which the blades creep resistance depends [13]. It is another reason for studying the variation of the crystal orientation in the area of the blade root near the connection with the selector.

The best method for such studies is the X-ray diffraction topography with the use of a divergent beam emitted from a microfocus X-ray tube and covering the whole sample surface that oscillates during exposure. By this method, a large area (from a dozen to several dozen cm^2^) of the blade with very low disorientation (arc minutes), as well as with the high disorientation (arc degrees), can be visualized in single topogram, which cannot be realized by other diffraction methods (e.g., EBSD or Berg–Barrett topography) [22,23]. In addition, the X-ray diffraction topography method allows analyzing the creation of the dendrite array during the dendrites’ lateral growth in blades [19]. At the end of the 20th century and at the beginning of the 21st century, the X-ray topography was used for the SX superalloy casts analysis; however, the studies were mainly concerned with the other issues. The grand effort was focused on increasing the resolution to visualize the single dendrites or groups of dendrites without focusing on the dendrite array in the blades of complex shape [24,25,26,27]. In those days, it was difficult to obtain the topograms from large sample areas of various disorientation arc minutes and degrees.

Recently, research on the dendritic array formation in the SX casts has been found to be extremely important [28]. Since the mechanisms of the dendrite growth from the selector to the root of the blade are related to the enlargement of the crystallization front cross-section, they have some features similar to the dendritic growth in the platforms [29].

The aim of the studies presented in this paper was to analyze the variation of the crystal orientation of the dendrite array in the blade root part located near the selector and to relate it to the process of the dendrites growth in the SX turbine blades made of CMSX-4 superalloy.

## 2. Material

The SX model blades (Figure 1a) used for the studies were produced by the directional Bridgman solidification of the CMSX-4 commercial superalloy. Five blades with an axial orientation of the [001] type were solidified using a spiral selector at withdrawal rate of 3 mm/min in an industrial (ALD Vacuum Technologies Inc., East Windsor, CT, USA) furnace at the Research and Development Laboratory for Aerospace Materials, Rzeszów University of Technology. The blades obtained at withdrawal rate of 3 mm/min are mostly used because, taking into account the production economics, they have the best creep resistance and are characterized by the good crystal structure perfection in comparison to the blades obtained at other rates [30].

Thin slices for examinations were cut-off from a part of the blades root, near the selector–root connection (S-R connection). Both slice surfaces of ABCD and A’B’C’D’, perpendicular to the blade axis Z, were carefully mechanical polished until a lamellar sample L (Figure 1a) of thickness m = 0.5 mm having two rhombus-shaped metallographic sections with diagonals AC = 20 mm and BD = 35 mm (AC = A’C’ and BD = B’D’) was formed. The blade axis Z was opposite to the withdrawal direction of Bridgman method. The shape of the lamellar sample L is presented in the upper part of Figure 1b.

## 3. Methods

The analysis of the crystal orientation and dendrite arrangement was carried out using scanning electron microscopy, Laue diffraction, and X-ray diffraction topography. A JEOL JMS-6480 scanning electron microscope was applied for dendritic structure observations using the backscattered electron technique (BSE). The macro-SEM images of the ABCD surface were created by the stitching of separate SEM micrographs. A computer processing was applied for better visibility of the dendrite arms direction. The processing combines a binarization of images and modification of contrast. The other methods are described below.

### 3.1. Laue Diffraction

The crystal orientation of the sample was determined by Laue diffraction using the X-ray diffractometer of an XRT-100 system provided by EFG Freiberg Instruments. The Laue patterns were recorded in back-reflection geometry. The arrangement of the sample L and X-ray Image Plate (IP) during exposure is presented in Figure 1b. The samples were mounted in a holder in such way that the DB section was parallel to the base line (BL) of the IP. The center of the surface area located inside the selector extension (SE) was exposed to an X-ray primary beam P (Figure 1b) of Cu_Kα_ radiation of about 0.8 mm in diameter. The SE is a fragment of the root limited by the cylindrical surface parallel to the axis of the blade (Z) and including the perimeter of the selector cross-section in the area near the S-R connection.

### 3.2. X-ray Diffraction Topography

**Figure 2:**
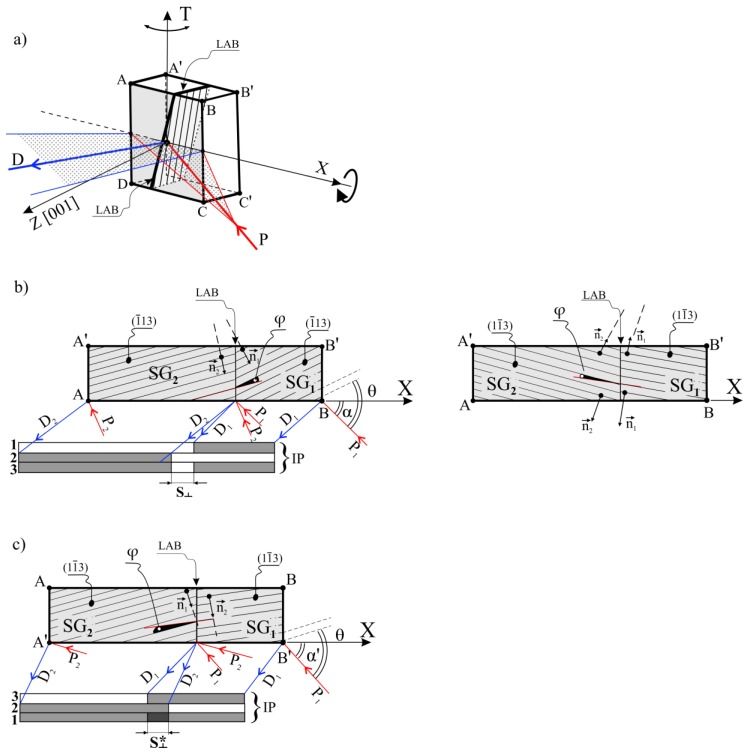
Scheme of the X-ray diffraction on the sample with two subgrains SG_1_ and SG_2_ (**a**) and formation of X-ray contrast originated for the ABCD and A’B’C’D’ sample surfaces using the $\bar{1}1$3 (**b**) $1\bar{1}$3 (**c**) reflections. 1,2—hypothetical partial layers of contrast originated from SG_1_ and SG_2_; 3—the complete contrast originated from the ABCD surface with a decreased contrast in the $S_{⊥}$ area and with increased contrast in the $S_{⊥}^{*}$ area originating from the surface A’B’C’D’; LAB—low-angle boundary; φ—angle of LAB misorientation; $\vec{n_{1}}$, $\vec{n_{2}}$—diffraction vectors. The sample thickness m is increased for figure clarity. The other symbols are descripted in the text.

The SX samples, initially the crystallographically oriented by the Laue method, were analyzed by X-ray diffraction topography. The microfocus X-ray Cu_Kα_ source provided by the Malvern Panalytical was used for X-ray topography. The topograms were obtained using the 113 and 002-type reflections during coupled sample and IP oscillation about the oscillation axis T. Figure 2a presents the geometry of diffraction from the ABCD surface of the sample with two subgrains SG_1_ and SG_2_, using the $\bar{1}13$ reflection. The topograms were obtained from the ABCD and A’B’C’D’ surfaces. The divergent primary beam (P, Figure 2a) covered the whole sample surface ABCD and formed a contrast in the topograms. The shape of the subgrains and various macroscopic defects originate from the local crystal misorientation, such as LAB, and the defects originated from the lattice distortions, such as the residual casting strain or inhomogeneity of the chemical composition, can be visualized on the topograms. Analysis of the pair of topograms obtained from the ABCD and A’B’C’D’ surfaces using the $\bar{1}13$ and $1\bar{1}3$ reflections allowed distinguishing the crystal misorientation defects and defining their localization. The details in the topograms obtained from the ABCD and A’B’C’D’ surfaces visualize the same defects that are similar in size to the sample thickness m. The defects are characterized by the contrast inversion; an increase in the intensity of the contrast from a certain area of the ABCD surface corresponds to a decrease in the intensity of contrast for this area obtained from the A’B’C’D’ surface.

### 3.3. The Principles of the Contrast Inversion in Topograms

The principles of the contrast inversion can be explained by the geometric relations between X-ray beams and the $(\bar{1}13$) and $(1\bar{1}3$) crystal planes. Since the thickness m of the sample L is small (0.5 mm), the topogram from the A’B’C’D’ surface should visualize the same defects as those visualized from the ABCD surface. Figure 2b,c shows the top view of the sample presented in Figure 2a. Let us analyze the formation of contrast from two subgrains SG_1_ and SG_2_ separated by an LAB (Figure 2). Before recording the lauegram, the sample was rotated by the δ angle (Figure 1b) in such a way that the line visualizing the crystal plane zone (ZL) and containing reflections 113 and 113 was parallel to the BL and the X axis (Figure 2a) as well as perpendicular to the oscillation axis T. The diffraction beam (D) was recorded on the IP. The crystal planes $(\bar{1}13$) in the SG_2_ subgrain are inclined relative to the crystal planes $(\bar{1}13$) in the SG_1_ subgrain by the angle φ (Figure 2b), which is the misorientation angle of the LAB. The P_1_ and P_2_ arrows present the paths of the incident beam P (Figure 2b) at various moments of the sample oscillation. The Braggs conditions are fulfilled if the incident beam falls on the SG_1_ along the P_1_ path or if the incident beam falls on the SG_2_ along the P_2_ path. When the sample oscillates, the Braggs conditions are fulfilled at various moments during changes of the sample oscillation angle. Since the crystal planes for the SG_1_ and SG_2_ subgrains are misoriented by the φ angle, the Braggs conditions for the subgrains are fulfilled at different sample oscillation angles and at different moments. The contrast formed by the beams D_1_ and D_2_ (Figure 2b) diffracted on the $(\bar{1}13$) planes of the SG_1_ and SG_2_ is recorded in the hypothetical layers 1 and 2 of the IP, respectively. The real topograms obtained on the IP (layer 3) consist of overlapped hypothetical layers 1 and 2. In the case of the real topogram obtained from the surface ABCD, there is decreased (brighter) contrast in the area marked as $S_{⊥}^{}$ because the diffraction vectors $\vec{n_{1}}$, $\vec{n_{2}}$ of SG_1_ and SG_2_ are divergent (Figure 2b—left side). The vectors are convergent when viewed from the A’B’C’D’ plane site (Figure 2b—right side). The topogram from the A’B’C’D’ surface was obtained after rotation of the sample by about 180° relative to the X axis, from the position when the topogram from the ABCD surface was obtained. In the case of the topogram obtained from the A’B’C’D’ surface, as a result of contrast shifts and the overlapping of hypothetical layers 1 and 2, the intensity of the contrast in the $S_{⊥}^{*}$ area is increased (darker), which is also related to the convergence of the diffraction vectors $\vec{n_{1}}$ and $\vec{n_{2}}$ (Figure 2c). The sample rotation around the X axis allows obtaining two topograms from ABCD and A’B’C’D’ surfaces for comparable incidence angles α and α’ (Figure 2b,c) that allow fulfilling the Bragg conditions.

## 4. Results and Discussions

**Figure 3:**
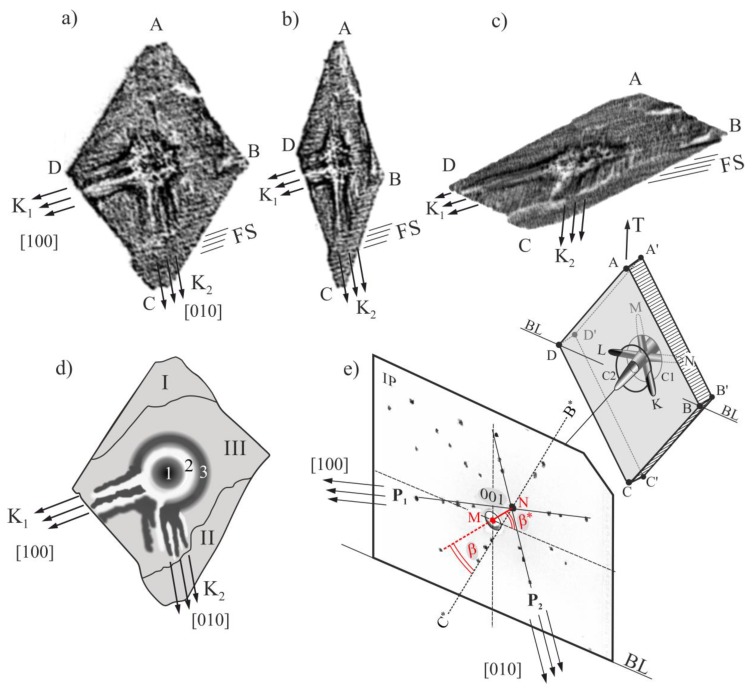
X-ray topograms obtained from the ABCD surface using reflections 113 (**a**), 002 (**b**), $\bar{1}\bar{1}3$ (**c**), and the scheme of the topogram from Figure 3a (**d**) as well as the Laue pattern recorded on Image Plates (IP) together with the location of the sample (**e**). The C1 and C2 circles belong to the planes A’B’C’D’ and ABCD.

Figure 3a–c shows the X-ray topograms obtained from the ABCD sample surface using reflections 113 (**a**), 002 (**b**), and $\bar{1}\bar{1}$3 (**c**).

**Figure 4:**
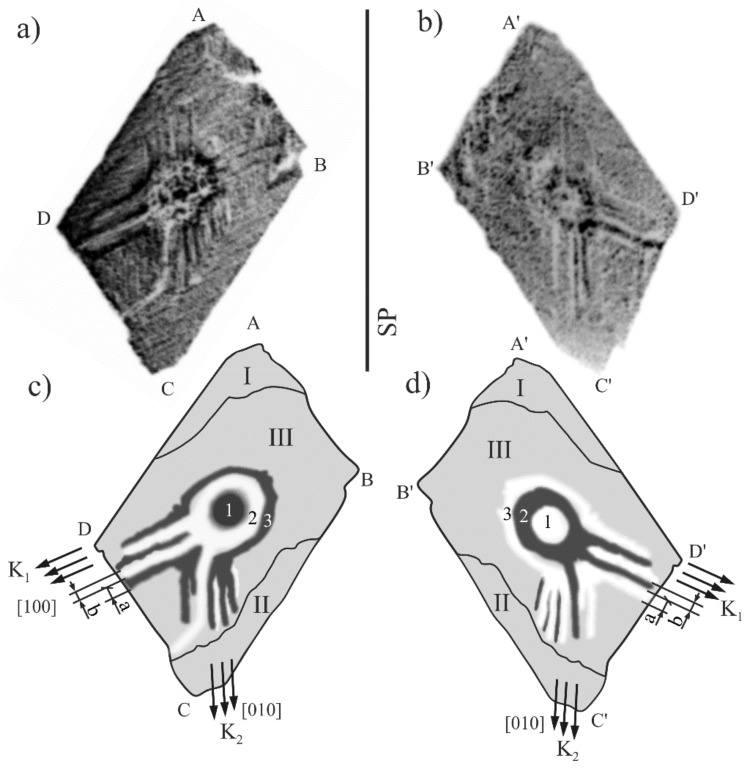
Topograms obtained from the surface ABCD (**a**) and A’B’C’D’ (**b**) using reflections $\bar{1}1$3 and $1\bar{1}$3, respectively, and their schematics (**c**,**d**); Cu_Kα_ radiation.

All topograms obtained from the ABCD surface visualize a subgrain structure containing generally three subgrains: I, II, and III (Figure 3d). The misorientation angle between the I and III subgrains is 1.2° degrees, and that between the II and III subgrains is 0.8° degrees. The method of misorientation determination was described in Ref. [31]. In addition, the pairs of the topograms obtained from the ABCD and A’B’C’D’ surfaces were recorded using the $\bar{1}13$ and $1\bar{1}3$ reflections (Figure 4a,b). The contours of the topograms (Figure 4a,b) are similar and symmetric with the SP mirror plane because the α and α’ incidence angles (Figure 2b,c) are similar due to the $(\bar{1}13$) and ($1\bar{1}3$) planes being tilted to the sample surfaces at similar angles.

The subgrains visualized in the topograms presented in Figure 3a–c are formed by fine (FS) and coarse (K_1_, K_2_) bands that are parallel to the [100] and [010] directions. The arrangement of these directions in the topogram was determined on the basis of the Laue diffraction pattern (Figure 3e). The system of the K_1_ and K_2_ contrast bands appear from the annular area 3 (Figure 3d and Figure 4c,d). In the central part of the topogram, corresponding to SE, the centrosymmetric areas of the increased and the decreased contrast are visible (Figure 3a–d). The areas of the increased contrast (darker) are the circular area 1 and the annular area 3. The annular area 2 with the decreased contrast (brighter) is located between them. The topogram obtained from the A’B’C’D’ surface shows similar-shaped areas, but the contrast is inverted in relation to the topogram contrast recorded from the surface ABCD. For example, the contrast of circular area 1 in Figure 4a has an increased intensity, and the same contrast area in Figure 4b has a decreased intensity. The effect of the inversion concerns the areas 1, 2, and 3 as well as the coarse bands K_1_ and K_2_. Figure 4 presents an exemplary pair of the a and b contrast bands belonging to the K_1_ coarse band. It means that the contrast from the ABCD surface presented in Figure 3a–c and Figure 4a has a misorientation character.

**Figure 5:**
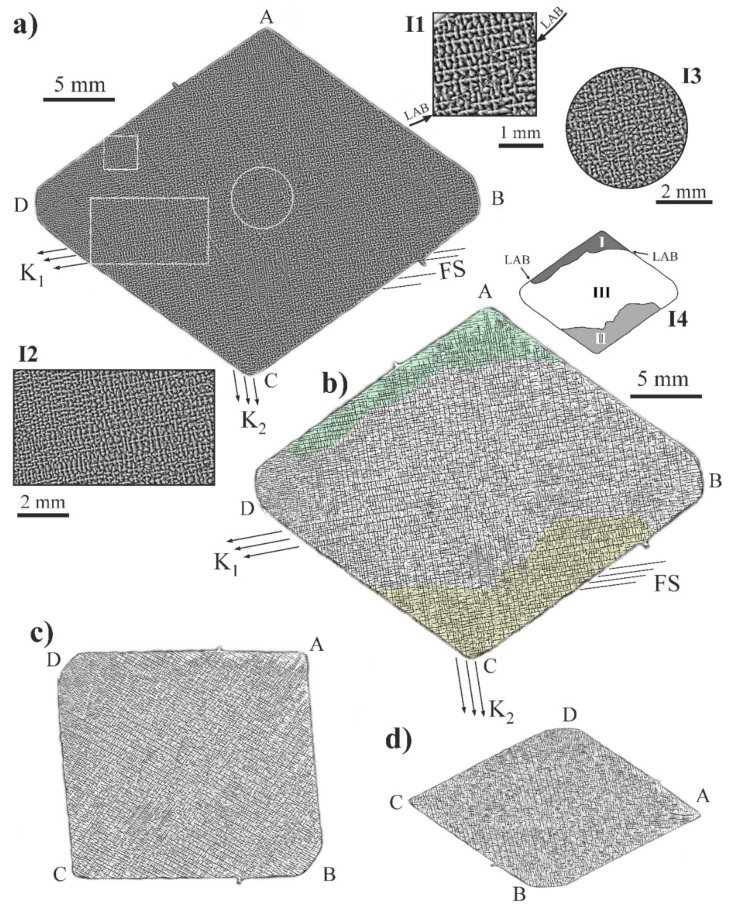
Image of dendritic microstructure obtained from the surface ABCD by SEM, BSE technique (**a**); and images obtained by computer processing of Figure 5a (**b**–**d**). The inserts I1, I2, and I3 are the enlargement fragments of Figure 5a. Insert I4 is the scheme of the subgrain structure.

In Figure 5a, the macro-SEM image of the dendritic structure obtained from the ABCD surface is presented. The selected areas of the macro-SEM image have been enlarged and presented as the inserts in Figure 5a (I1, I2, and I3). Figure 5b was obtained by computer processing the macro-SEM image from Figure 5a without resizing. Insert I4 presents the schematic shape and arrangement of the subgrains I and II. Figure 5c,d was obtained by further computer image distortion and resizing. The SEM images show a typical dendritic structure with a four-petal flower morphology of secondary dendrite arms (Figure 5a—inserts I1, I2, I3). The dendrites are arranged in arrays that differ depending on the sample areas. The differences in the dendrite array may be observed e.g., between the subgrains I and III separated by the LAB (insert I4), between which the differences in dendrite arms arrangement are observed (insert I1); these are better visible in the Figure 5b. In certain areas, the secondary dendrite arms create long straight chains of K_1_, K_2_, and FS types (Figure 5a,b, insert I2). The chains of the secondary dendrite arms arranged parallel to the contrast bands FS, as well as to the K_1_ and K_2_ directions, are more visible in Figure 5b–d. However, without analyzing X-ray topograms, the described defects would be difficult to find in the SEM images of the microstructure. The shape of the area located in the center of the sample is similar to circular and corresponds to the SE, which is more visible in Figure 5c. Additionally, in the central part of the sample, the long dendrite chains are rarely observed (insert I3), which is better more in Figure 5b–d. Similar chains are described in Ref. [7,19]. The contour of the macro-SEM images from (Figure 5a,b) differ little from the contour of the topograms presented in Figure 3 and Figure 4, because the crystal planes fulfilling the Braggs conditions are inclined relative to the ABCD or A’B’C’D’ surfaces.

**Figure 6:**
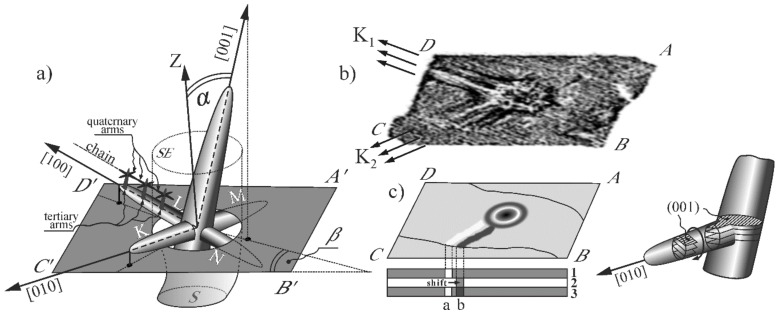
Arrangement of subsequent dendrite arms during the start of the primary arms growth from the selector to the root (**a**) and the topogram obtained by the computer processing of the topogram from Figure 3b (**b**) as well as the scheme of creating one of the contrast bands of K_2_ type formed by crystal planes rotation (**c**). The α angle is enlarged for the figure clarity.

The directions of the K_1_, K_2_, and FS bands visualized on the topograms are compatible with the arrangement of the dendrite arms chains visible in Figure 5a,b. Therefore, it was concluded that these bands are parallel to the chains of the dendrite arms and also that the crystal orientation of the chains are [100] or [010] type. Figure 6a presents the scheme of lateral growth of the secondary dendrite arms during the start of the primary arms growth from the selector to the root and exemplary subsequent dendrite arms arrangement. The tertiary dendrite arms from which the quaternary arms grow in turn grow from the secondary dendrite arm L visible above the A’B’C’D’ surface (Figure 6a). These tertiary arms are arranged in the chains of crystallographic orientation inherited from the secondary dendrite arm L (Figure 6a). The K_1_ and K_2_ coarse bands visible in the topograms are related to the chains of quaternary dendrite arms presented in Figure 5. These arms grow from tertiary dendrite arms (Figure 6a), which in turn grow from the secondary arms K and L. Based on the topograms presented in Figure 3a–c, it may be concluded that the L and K arms spread from the annular area 3 (Figure 3a–d). Therefore, it was concluded that the K_1_ and K_2_ bands direction corresponds to the direction of the secondary arms created by the lateral growth from the primary arms growing from the selector toward the root. That types of arms grow at a significantly higher rate in comparison to the growth rate of the primary arms, as described in Ref. [19].

The four types of the secondary dendrite arms, marked in Figure 6a as K, L, M, and N, can grow laterally from the primary dendrite arm (Figure 6a). However, on the topogram of the 002 reflection, only the bands parallel to the K and L arms are visible (Figure 6b). The primary arms inclination relative to the A’B’C’D’ surface, defined by the position of 001 reflection in the topogram, shows that the arms K and L begin to grow in the SE as first, ahead of the M and N arms (Figure 6a). Therefore, such dendrite arms can be called leading arms. The earlier growth of these arms is related not only with the α angle (Figure 6a), but also with the β angle, which is the angle between the B’C’ edge of the root and the projection of the [001] direction on the A’B’C’D’ surface perpendicular to the blade axis Z (Figure 6a). In the analyzed case, the α angle is 9° and the β angle is 35°. The values of the α and β angles can be easily determined from the Laue pattern using QLaue software. The α angle is an angular measure of the distance between the center of the lauegram (point M, Figure 3e) and the reflection 001 (point N, Figure 3e). The β angle is the angle between the extension line MN and the B*C* line parallel to the B’C’ edge of the root.

These arms are responsible for the creation of increased and decreased contrast bands K_1_ and K_2_ in the topogram (Figure 6b). The bands formed as a result of a shift of the contrast (Figure 6c). In turn, the shift corresponds to the rotation of the crystal planes (001), which means the rotation of the crystal lattice of the adjacent chains of dendrites arms visualized in Figure 5. This rotation concerns the crystal lattices of tertiary and quaternary arms, which grow from the secondary leading arms of K and L type. As it can be seen in Figure 3a–d and Figure 4a, the bands of the K_1_ and K_2_ type spread from the outer circular area 3 of the increased contrast (Figure 3d). Therefore, it may be concluded that the leading arms of K and L type (Figure 3a–d) spread from area 3. It means that the crystallization front starts to extend by the lateral growth of the leading secondary dendrite arms growing from the primary dendrite arms located on the perimeter of the SE section.

**Figure 7:**
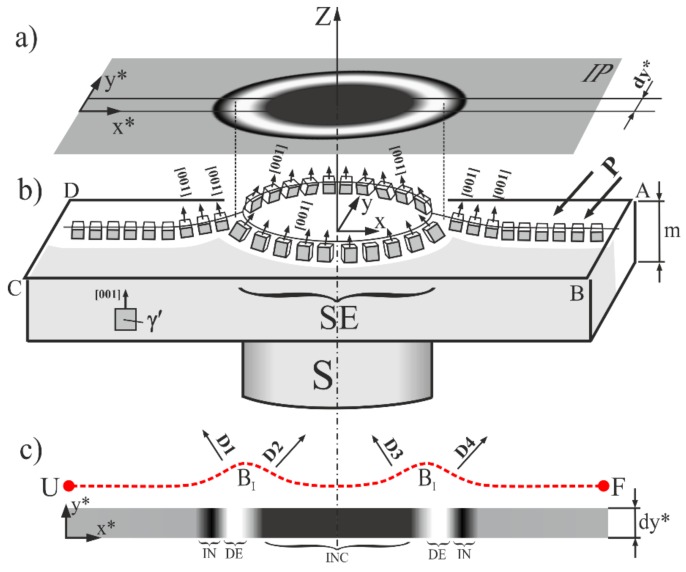
Scheme of the orientation contrast created by the SE region of the root (**a**) and the distribution of the [001] orientation of the γ′ phase crystals (**b**) as well as the distribution of the contrast intensity in the thin d_y_ band of the Image Plate (IP) (**c**). The angles of the slope of the vectors [001] are enlarged for the figure clarity; X*,Y*—axes on the IP; X,Y—axes on the ABCD surface.

The effect of the creation of circular and annular contrast areas in the topograms is related to the local crystal misorientation and may be explained by a simple scheme, as presented in Figure 7. The following discussion were carried out based on the analysis of the topograms obtained for the 002 reflection (Figure 3b). A homogenous contrast recorded in the topograms and represented by a grey homogeneous area on the IP (Figure 7a) indicate that the crystal orientation in this area is the same. For simplicity, it was assumed that the diffraction of X-rays takes place on the crystals of the γ′ phase only because the fraction of this phase is the highest: about 70% [1]. When the γ′ crystal’s orientation changes in a certain area—for example, in the SE area (Figure 7b)—the intensity of contrast recorded in the IP for the neighboring area is higher or lower due to the overlapping or separation of the inclined diffraction beams (D_1_–D_4_). This results in the local increasing or decreasing contrast in the topograms described for the LABs in the Methods section (Figure 2).

Two types of γ′ crystals’ misorientation can be determined in the SE area—the convex (corresponding to beams D_1_ and D_4_) and the concave (corresponding to beams D_2_ and D_3_) (Figure 7b). Therefore, two types of contrast shift can be observed: outside and inside the Z-axis direction of the blade (Figure 7c). A fragment of the IP with width d_y_ is shown in Figure 7c to visualize the shifts. The outside shift increases the contrast in the IN areas and decreases in the DE areas, while the inside shift increases the contrast in the INC area of the SE area and decreases the contrast in the DE area. The local bending of the UF section on Figure 7c describes a distribution of the [001]γ′ crystallographic direction along the X axis, which is perpendicular to these sections. The example shown in Figure 7 presents only symmetrical deviation from the growth direction Z, but in most cases, the symmetry of the contrast slightly differs from centrosymmetric, similar to that in the presented topograms (Figure 3b,c and Figure 4a,b). This is related to the inclination of the crystal planes, which fulfills the Bragg conditions in relation to the analyzed surface described by angles α and β (Figure 6). The shape of the LB section may be similar to the shape of the solidification front with the assumption that the primary dendrite arms always grow in the [001] direction, which is always perpendicular to the crystallization front.

The character of the bends of the UF section described by the local maxima B_1_ and B_2_ (Figure 7) are similar to the isotherm bends described by the cellular automaton finite element (CAFE) simulations in Ref. [32] for the platform region of the blade, and these are called “wave-shaped”. The platform region has one common feature with the SE area—a step increase of the blade cross-section.

The misorientation defects visualized in the topograms by the K_1_ and K_2_ bands may be the cause of LABs creation in the areas of the root located near the selector and in the next step inherited by other parts of the root and by airfoil crystallizing later [18]. It is the reason why the location of the K_1_ and K_2_ bands and the mechanism of defects creation inside them are extremely important.

## 5. Conclusions

1. The crystal orientation of the primary dendrite arms in the selector extension area of the root determines the areas and directions of lateral growth of such secondary dendrite arms in root, which cause the creation of the low-angle boundary type defects. These arms can be named the leading arms because in the root, they grow laterally at first.
2. The lateral growth of the leading dendrite arms in the root is related to their crystal lattice rotation, which is the reason for the low-angle boundary type defects creation.
3. In the selector extension area of the root near the connection with the selector, the spatial distribution of the [001]γ′ crystallographic direction has a complex wave-like character, which is approximately symmetric to the blade axis Z.
4. The leading secondary dendrite arms start to grow from the primary dendrites located on the perimeter of the selector extension area.
5. The spatial distribution of the [001]γ′ crystallographic direction in the selector extension area may reflect the shape of the crystallization front with the assumption that the primary dendrite arms always grow in the [001]γ′ direction, which is always perpendicular to the crystallization front.
